# Dynamic community evolution analysis for performance optimization in large scale RFID networks

**DOI:** 10.1038/s41598-026-43260-x

**Published:** 2026-05-13

**Authors:** M. Thurai Pandian, H. Anwar Basha, Tanvir Habib Sardar, Sk Mahmudul Hassan

**Affiliations:** 1 School of Computing, SRM Institute of Science and Technology, Tiruchirapalli, Tamil Nadu India; 2 Department of Computer Science and Engineering (Artificial Intelligence and Machine Learning), Rajalakshmi Institute of Technology, Chennai, Tamil Nadu India; 3 Department of Computer Science and Engineering, School of Engineering, Dayananda Sagar University, Bangalore, Karnataka India; 4 Manipal Institute of Technology Bengaluru, Manipal Academy of Higher Education, Manipal, 576104 India

**Keywords:** RFID, NCEA, Complex networks, Cluster head, Evolution framework, Weak and strong events, Engineering, Mathematics and computing

## Abstract

Handling Large scale RFID network is most important for Tracking and Locating objects in the Intelligent Transportation System and IoT based RFID networks. This research work focuses to build the road map with RFID support without interference of satellite. The cluster-based RFID network can be supported to enlarge the RFID network. In this paper, proposes the Novel Community Evolution Analysis (NCEA) method for selecting cluster heads, designed to tackle both strong and weak events within the network. The traditional clustering method may miss some weak events occurrences and its leads to impact the efficiency of cluster head selection of the cluster network. To identify this problem, our method incorporates the required strong and weak events. This proposed method concentrates the energy management and cluster head selection management accurately. Also, the cluster head selection may happen through high energy node and close neighbor node to all the participated nodes in the cluster. The cluster head selection can be happened by different events such as form, disappear, shrink, expand, split, merge. The NCEA approach demonstrates an accuracy of 98%, a vulnerability rate of 20%, a success rate of 89%, a latency of 11.4 s, and a throughput of 93%. These results highlight the effectiveness of our method in enhancing the energy efficiency and overall performance of large-scale RFID networks.

## Introduction

RFID is a well-recognized technology used for the purpose of identifying various types of things. This technology is rapidly advancing and has the potential to provide substantial financial profits in several businesses and the digital realm. RFID plays a key role in the IoT (Internet of Things) application domains such as intelligent transportation, Tracking objects, security and access control. RFID systems have the capability to register things and facilitate tasks like counting and monitoring moving and stable objects. The design of RFID network protocol is rather intricate. The restriction of power supply limits the design of any RFID network. Furthermore, reducing power consumption during the energy utilization process is a crucial concern in the planned use of the RFID network protocol. Moreover, RFID systems should focus on certain factors, such as reliability, scalability, and acknowledgments^1^^,^^2^. Low power RFID readers are indispensable to ensure dependable and secure communications. The communication methods using the most resources to transfer information from the node to the base station cause some traffic. Gaining knowledge of utilizing the direct transmission protocol to connect directly to the base station and reduce the load on the communication mechanism is crucial^3^. In this posture, under these scenarios, readers are often placed at a distance from the base station, which limits energy being reflected back towards the reader. The primary reason for a high consumption of energy in this process is due to the action of separation. Therefore, low-capacity batteries encounter trouble returning the signals reasonably well to the readers. A few authors had suggested methods for improvements the MAC and network layers^4^. However, an obvious issue occurs when there are several nodes trying to take the lead and compete on leading other nodes in the network. Clustering strategies are fundamental techniques for addressing challenges in networks^5^. In our current design, each reader examines the data and transmits it to the base station for tag identification, which reduces the efficiency of the readers. The network will be designed using the cluster method to improve the efficient group of readers in the RFID system. Each cluster will be determined based on the most accurate customer and the group of customers performing the same actions^6^. The cluster will amalgamate bases all relevant client data before the request for connection is submitted. The original Clustered RFID computation exhibits higher performance levels while maintaining management over the large-scale RFID framework. This work will focus on improving the analysis based on community analysis, in particular, analyzing the evolution of communities^7^. The analysis provides critical implications for the three areas: (1) Determine when there is a significant change in patterns of interaction; (2) understand the underlying structures of complex networks; and (3) predict future trends in networks^8^. Sometimes when observing a large-scale RFID system with a large set of readers, we are concerned with a subset of tags-specific tags. We refer to these tags as key tags, thus ignoring detecting tags beyond those deemed key^9–11^. As an example, employees might be required to take inventory of goods from a specific category in a mall with tens of thousands of items. The tags associated with those goods would be identified as important tags. The remaining tags can be identified as inconsequential tags or out-of-interest tags. In contrast, a retailer with limited bandwidth may pay more attention and focus on items that are of higher value (key tags) than items that are less expensive; with key tags the frequency of inspecting main item would be much greater than inspecting tag associated to less expensive items. In both scenarios the more efficient tagging process of providing key tags to the readers would only allow readers, tagged with key tags to zoom in on and engage with the associated areas to ensure efficient key tag management. The average supply chain construction process might benefit from an aliased RFID process into its supply chain system. However, localization and security issues prevent it from being commonly used^12^.

The goal of this assessment is to reveal the practical use of RFID technology in the construction industry’s supply chain processes with respect to limitations in localization. The objective of the investigation is to demonstrate how RFID can provide better and useful solutions with other alternative technologies such as IoT, and solid secured processes^13^. We examine the shortcomings of RFID localizations and talk about the difficulties preventing the construction industry from using RFID on its own. In order to minimize energy loss and enhance network efficiency, it is necessary to use a clustering mechanism that incorporates efficient cluster head selection. This mechanism should effectively manage complicated networks while minimizing energy consumption. This is to develop and implement a comprehensive framework for dynamic community evolution analysis in large-scale RFID networks, which is aimed at optimizing energy efficiency and network performance through innovative cluster head selection and enhanced management of both strong and weak events.

Building RFID based Route map is used to track the location of the vehicles without any support of satellites. Global Positioning System (GPS) will support in the non-crowded building area and secure high cost. Single source error can affect the entire system. Cluster based RFID network can be supported for enlarge the RFID network and support to route map construction without compromise the efficiency. In this paper Novel Community Evolution Analysis (NCEA) algorithm is proposed for form the clustered RFID network. This proposed algorithm can be supported for large scale RFID network without compromise the network efficiency. The existing GPS system cannot tolerate the single source (satellite) failure. The cost of launching satellites are very expensive compare than RFID network.

### Contributions of the work

- Develop strategies to optimize both performance and efficiency in large-scale RFID networks, ensuring robust operation and extended network lifespan.
- Adopt a novel approach to cluster head selection based on community evolution analysis. This method aims to effectively manage extensive RFID networks while maintaining peak performance levels.
- Propose and apply a novel concept of weak events, defined by the degree of community overlap and membership. This innovation aims to refine the comprehension of community dynamics and improve the conventional frameworks for community evolution that are dependent on events.
- Analyze the performance of the network by the network parameters and produce the better performance than the kg**-DFSA and KMCA.**

In this paper Section "Introduction" describes the state of the art of the RFID networks. Section "Related Works" describes the Related works based on the RFID cluster networks. Section "Proposed methodology" describes the Clustering and Cluster head selection of the Complex RFID network. Section "Results" represents the performance analysis of the proposed novel Community evolution analysis algorithm (NCEA) by comparing it with existing methods such as kg-DFSA and K-Means Clustering algorithm (KMCA). Section "Conclusion" closed with a conclusion.

- The novel aspect of this research is, the traditional RFID network optimization focuses on static or single-state community structures. This system may be unique in that it can accommodate variations in network density and signal interference while dynamically identifying and adapting to shifting community patterns within large-scale RFID networks. Static or single-state community structures are the main focus of traditional RFID network optimization.
- A novelty of this case may be a new energy-efficient algorithm designed for larger networks that can distribute energy consumption evenly through the network to reduce energy consumption without a decrease in performance.

## Related works

RFID is a key technology for IoT and big data. It facilitates pairwise object information transfer, which creates a lot of data when the object is transmitting. There have been multiple research studies and methodology of a few of the existing options will be outlined. Based on article^14^, there is a kg-DFSA RFID anti-collision method named kg-DFSA that allows the reader to have information to predict tag positions based on past knowledge of the reader. In kg-DFSA the tag identification process is categorized in two sets, the initialization phase and identification. In the initialization phase the reader uses an enhanced K-means clustering algorithm while using a tag counting algorithm. The K-means clustering algorithm bins the tags into K clusters based on the RN16 value of the tag and the tag counting algorithm estimates the number of tags.

The success rate is 50%, the system efficiency is 65%, and the identification time is 28. The Signal strength measurement is used in^15^ to get the estimated distance via the utilization of the Received Signal Strength Indicator (RSSI) technique. The server employs the K-Means Clustering technique to partition the observation area into clusters. The result of this categorization is the identification of concentrated areas (traffic) and dispersed areas to establish the coverage zones of each access point.

The study^16^ used clustering approaches to determine the clustering strategy that yields the highest performance in the given circumstance. This work’s primary contribution is a precise positioning system designed for extensive areas. In our study, we demonstrated a decrease of 38.16% in processing time and 58.39% in classifier model size using the suggested hierarchical technique.

The HPSO-RNP approach, proposed in^17^, combines particle swarm optimization with K-means clustering and virtual forces to optimize RNP. It excels in terms of the quantity of readers, interference management, power efficiency, and load balancing when it comes to designing RFID networks. The superiority of the LANDMARC system, which is based on dynamic active RFID calibration, for indoor locating in complicated environments was stated in^18^. The primary obstacle in designing an RFID network is determining readers’ ideal positioning and settings to fulfil the RFID system’s fundamental criteria, including coverage, load distribution, and reader interference^19^.

A new optimization method, the self-adaptive cuckoo search (SACS) algorithm, is designed to handle difficult problems. This technique allows for the dynamic adjustment of parameters in real time for the cuckoo search (CS) process. The self-adaptation phenomenon enhances the flexibility of the evolutionary algorithm, bringing it closer to the dynamics of natural evolution. In^20^, it offers a way to dynamically choose the cluster head while accounting for each RFID reader’s power and connectivity. Fuzzy Logic is the basis for choosing a new cluster head in dynamic mode. According to these data, a node has a 91.8% chance of becoming a CH at the energy level of 0.443 and centrality of 0.809.

A decomposition-based enhanced multi-objective brainstorming optimization method (IMBSO) is proposed in^21^. The search is more effective when decomposition divides the multi-objective optimization problem into multiple smaller problems. This allows all objective functions to be optimized at the same time. BF Search, a Bloom filter-based tag searching method with two stages—the target tag verification phase and the non-wanted tag deactivation phase—is proposed in^22^. To eliminate their interference during the non-wanted tag deactivation phase, we offer a composite filter vector that can deactivate local tags that are not included in the target tag collection. A reliable and effective missing key tag identification (ERKI) protocol with a filtering and verification mechanism is proposed in^23^. RFID network planning, swarm intelligence optimization methods, smart factories, and RFID technologies are reviewed in^24^.

The elements influencing RFID network performance and the path of improvement for swarm intelligence optimization methods are also investigated. In^25^ In order to increase the utilization of polling vectors, create a double polling mode that allows two tags to be queried simultaneously. The aforementioned surveys provide a comprehensive grasp and extensive knowledge of the concept of clustering and its methodologies. Nevertheless, they have certain constraints when it comes to discussing the environmental context, analyzing parameter settings, and incorporating modern clustering algorithms that align with the prevailing trend of RFIDs. Moreover, conventional approaches are unprepared to handle weak occurrences, which might be characterized as events prompted by little changes in the community. These events are not deemed evolutionary since they are subject to the stringent limitations of strong events.

Traditional approaches often provide poor accuracy when it comes to discovering evolutionary events. The Weak-Event based Community Evolution Method (WECEM) framework’s basic idea and operation for weak event mining in the community evolution of dynamic complex networks. Events are divided into strong and weak events by WECEM. The continuity of dynamic communities in complex networks is assessed using community membership degree and community overlapping degree. The WECEM framework examines each community at successive timestamps to determine the community overlapping degree and community membership degree, respectively, and then uses these two measurements to identify distinct events^26^. Table 1 represents the some of the related works based on the RFID cluster network.

**Table 1:** Related works.

Ref	Proposed method	Findings	Limitations
^14^	Proposes an **efficient tag grouping** algorithm using **improved K-means clustering** for IoT-based RFID systems	Introduces clustering-driven DFSA that improves efficiency in IoT RFID environments	Requires additional computation for clustering - Assumes availability of signal strength data
^27^	Develops a **Dual-Response Collision Tree (DRCT)** protocol to accelerate tag identification in large-scale RFID networks	Introduces a novel dual-response tree structure that accelerates tag identification for large-scale RFID systems	Requires hardware capable of handling dual simultaneous responses - Slightly increased complexity at the reader side
^28^	RFID itinerary-based security and detection model	Introduced a **secure RFID itinerary model** improving both detection accuracy and system integrity	Limited scalability in high-density environments
^29^	Network routing and performance optimization framework	Delivered an **evolutionary performance analysis** of **PEIS with GPS and AOMDV-SAPTV**, establishing benchmarks for QoS improvement	Energy consumption not deeply optimized
^30^	Distributed trajectory clustering	Introduced a scalable, uncertainty-aware RFID clustering method for cloud environments	Dependent on cloud infrastructure and parallel frameworks - Computationally heavy for very large trajectory sets
^16^	Hybrid clustering and hierarchical classification	Developed a precise hybrid clustering–classification model for RFID-based indoor localization	- Requires training data for classification - Sensitive to environmental interference
^31^	Statistical estimation at the physical layer	Proposed a novel physical-layer estimation framework improving scalability and accuracy in RFID systems	Assumes ideal channel conditions - Limited applicability to small-scale systems
^32^	Signal-based clustering (RSSI correlation and time-series processing)	Presented an RSSI-based clustering technique to locate lost RFID-tagged items effectively	RSSI instability may affect accuracy - Limited range for active tags

## Proposed methodology

**Fig. 1:**
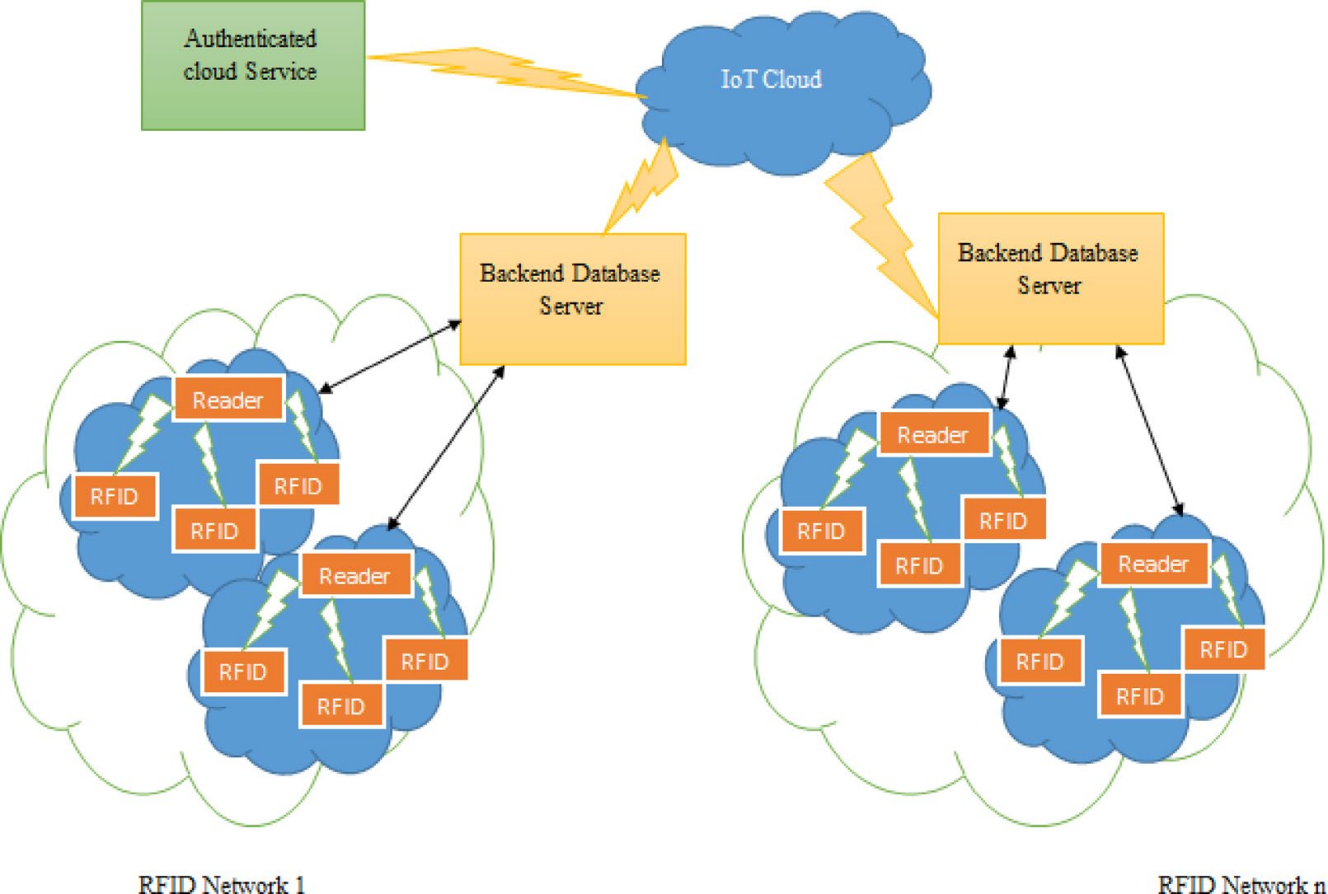
RFID-based distributed system architectures.

In this paper, we provide a distributed system architecture based on RFID, which forms the basis of our suggested methodology. The network architecture of our lightweight, anonymous RFID authentication method is shown in Fig. 1. Each of the various RFID networks that make up the RFID-based distributed system design is further divided into a large number of clusters. Here, we introduce a distributed IoT system architecture based on RFID, which serves as the framework for our suggested plan. The RFID-based distributed IoT system design is separated into many RFID networks, each of which can be further subdivided into multiple clusters.

Each cluster is equipped with an integrated RFID-tag reader, while each RFID network is associated with its own backend database server. Hence, the proposed design consists of four crucial components, (i) a reader (R) (ii) a server, referred to as (Server), and an RFID-tag, labeled as Tag (T) Both the RFID tags and the readers must be registered with a designated back server. Under such circumstances, both Tag (T) and reader (R) will get security credentials from the Server. Additionally, it is necessary for each Server to complete the registration process with ACS (Authenticated cloud service). ACS facilitates mutual authentication and the shared security credentials or establishing a secure channel between two backend database servers that want to connect with each other. Currently, we enable Tag (T) to transition across clusters and even between different networks. When Tag(T) communicates with a reader (R) of a certain cluster, reader (R) must engage with the relevant backend server to identify Tag (T). Only the specific backend server that has registered Tag (T) may recognize it. Therefore, the Tag(T) transitions from its original network (to which it belongs) to a different network. Under such circumstances, it is presumed that both the database servers are capable of communicating with each other over a secure channel, which has been constructed to facilitate ACS. Thus, only the specific backend server associated with Tag(T) may understand the initial identity and movement of Tag (T).

### Clustering process and cluster head selection

**Fig. 2:**
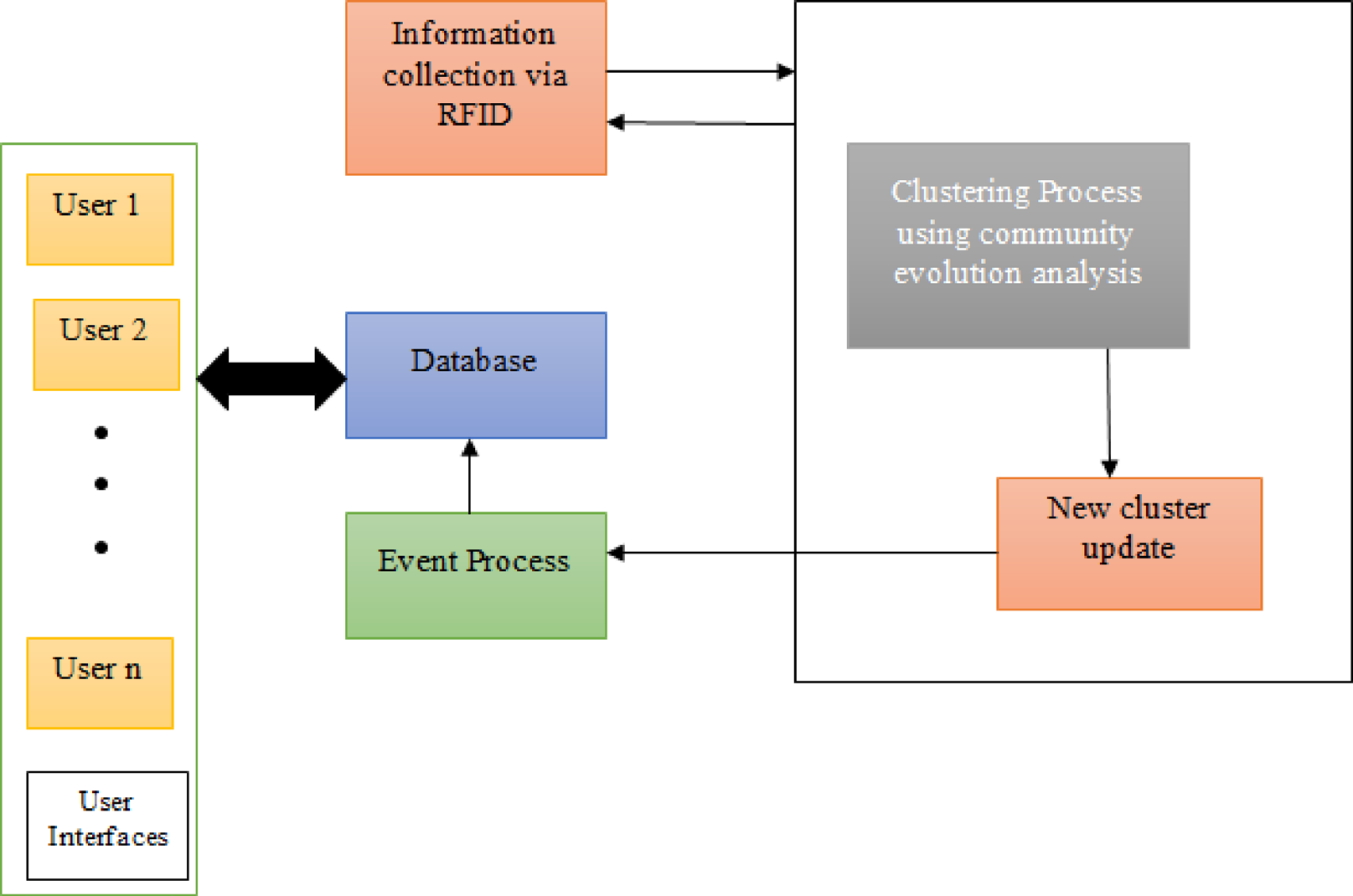
Clustering and cluster head selection process.

Within the RFID network, the nodes were organized into groups, with each node relying on the others for effective communication. A cluster is formed by a set of nodes, with one node being chosen as the cluster head (CH) as shown in Fig. 2.

The cluster-head node has several attributes, including.

- The node must be positioned centrally within the cluster and possess strong interconnectivity with other nodes.
- The chosen node must have a higher energy level than the others. Furthermore, if the cluster head cannot fulfill its role in leading the communication, another adjacent node with similar characteristics to the current cluster head will assume the responsibility of leading the communication. The community evolution analysis approach is capable of identifying the subsequent cluster head.

The group leader updated the data in the information base located on the user. The obtained tag data efficiently guided the dataset within the shortest timeframe, necessitating improved development direction. The purpose of the tags is to enhance the display via clarity and ensure security. In order to implement the RFID strategy successfully, it is crucial for the readers to aggregate efficiently, generate tags, and prioritize optimal routing circumstances.

### Novel community evolution analysis-based cluster head selection

In the context of extensive and intricate RFID dynamic networks, the formation of links between nodes leads to a gradual evolution of community structures. Hence, altering community structures over a very little timeframe becomes challenging. Algorithm 1 represents the Weak and Strong Community Evolution Detection. Hence, although major transformations are rare, it should not be assumed that little changes do not occur regularly throughout the intervals between major changes. Consequently, if conventional techniques are only intended to identify significant, overarching transformations in intricate networks, they fail to discover smaller, less impactful occurrences. This is the driving force for our suggested weak-event-based method, where the notations are shown in Table 2.

**Table 2:** Denotation of symbol and description.

Symbol	description
$rem,fo,diapp,exp,sh,sp,mer$	The community set consists of events such as remaining, forming, disappearing, expanding, shrinking, splitting, and merging
${w}_{sh},{w}_{exp},{w}_{sp},{w}_{mer}$	The community sets exhibit weak shrinkage, weak expansion, weak splitting, and weak merging occurrences, respectively
$over$	Community overlapping degree
$mem$	Community membership degree
${com}_{n}^{q}$	Community at time n with label q
${com}_{n+1}^{q}$	Community at time n + 1 with label q
$\alpha$	Determining community existence
$\beta$	Judging community size
$\gamma$	Judging multi-community changes

**Algorithm 1:**
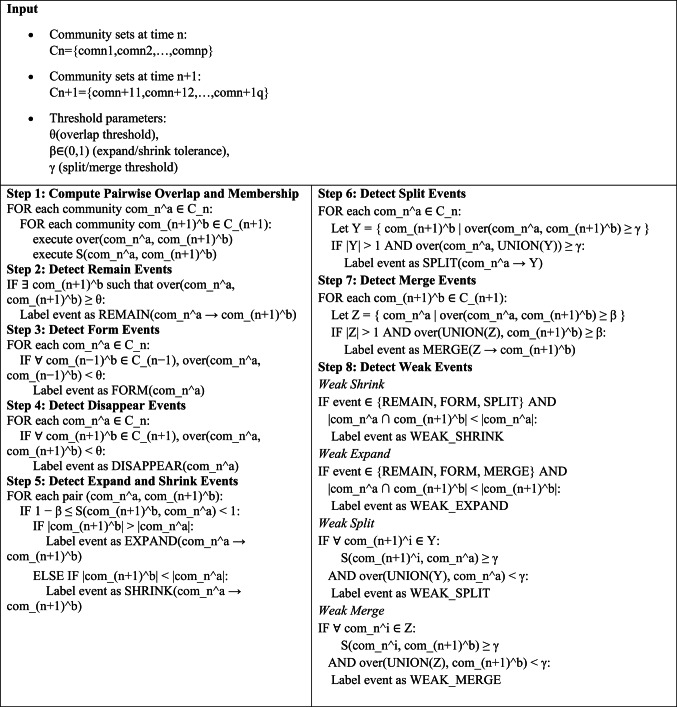
Weak and strong community evolution detection

$Dynamic\_Community$: Let ${com}_{n}=({\partial }_{1}^{n},{\partial }_{2}^{n},\dots {\partial }_{N}^{n})$ denote a community at time n, where ∂ represents the RFID node in ${com}_{n}$ and ∂ represents the node in ${com}_{n}$. Some events may happen when time t to n + m in the context of ${com}_{n}$. $\{{com}_{n},\Delta {B}_{1},\Delta {B}_{2},\dots .\Delta {B}_{m}\}$ signifies a vibrant community, as shown by ${com}_{n:n+m}$. The network can be formed as ${com}_{n+m}={com}_{n}+\Delta {B}_{1}|+\Delta {B}_{2},\dots .+\Delta {B}_{m}$, from time n to n + m which denotes the events occurring in ${com}_{n}$. Every time when the network changes its structure, the new network can be formed. We call the event as “strong_event.". At the time step n + 1, the community ${com}_{n+1}^{2}$ divides into two smaller communities due to changes in the interactions between nodes 10 and 11 and other nodes. At the same time, the structure of ${com}_{n+1}^{1}$ stays constant, indicating that ${com}_{n+1}^{1}$ is considered to persist as an occurrence. This network gains nodes 13 and 14 at time n + 2, and node 14 connects to nodes 3, 4, and 6. Simultaneously, node 13 forms a connection with node 11. The sizes of community ${com}_{n+1}^{1}$ and ${com}_{n+1}^{3}$ increase as a result of these adjustments. At time n + 3, the community ${com}_{n+1}^{3}$ decreases in size due to the removal of node 1 from ${com}_{n+1}^{3}$. At time n + 4, community ${com}_{n+3}^{1}$ decreases in size as node 1 leaves ${com}_{n+3}^{1}$. At time n + 4, the community ${com}_{n+4}^{3}$ ceases to exist entirely since its nodes no longer have any connections with other communities. The connections between nodes 4, 5, 7, and 12 get stronger at time n + 5 to such an extent that it results in the fusion of ${com}_{n+4}^{1}$ and ${com}_{n+4}^{2}$.

Remark 1: Measurable change in non-real time evolving communities in complex networks might occur slowly due to their specific properties. Simultaneous evolutionary processes may occur in comparable groups.

$Weak\_Event$: Subtle alterations in the community set off a weak event. While strong events may not acknowledge their presence, weak events such as $Weak\_Shrink$, $Weak\_Expand$, $Weak\_Split$, and $Weak\_Merge$ events may happen concurrently with strong events.

At time n, the community ${com}_{n}^{2}$ divides and gives rise to the communities ${com}_{n+1}^{2}$ and ${com}_{n+1}^{3}$, which are understood at time n + 1. Concurrently, node 8 is present in ${com}_{n}^{2}$ and ceases to exist in the network at time n + 1. It is evident that a gradual reduction event takes place from n to n + 1 in ${com}_{n}^{2}$ and ceases to exist in the network at time n + 1. A mild shrink event may be detected in ${com}_{n}^{2}$, transitioning from n to n + 1. At time n + 1, communities ${com}_{1}$ and ${com}_{2}$ merge, and node 15 also joins. This results in the manifestation of a weak expanding phenomenon. At time n, the communities ${com}_{n}^{2}$ and ${com}_{n}^{3}$ are associated with ${com}_{n}^{2}$, and nodes 8 and 9 vanish at time n + 1. Therefore, it may be inferred that a minor division event takes place at time n + 1 in ${com}_{n}^{2}$ at time n. At time n, the community ${com}_{n}^{1}$ and ${com}_{n}^{2}$ belong to community ${com}_{n}^{1}$ at time n + 1. Therefore, a very insignificant merging event takes place at time n + 1 between communities ${com}_{n}^{1}$ and ${com}_{n}^{2}$ 2, which were present at time n.

$Community\_Overlapping$: The ratio of the number of nodes that are common to both communities to the total number of nodes in either community is used to calculate the degree of community overlap between communities *comna* at time n and *comn* + 1*b* at time n + 1.

1 $$over\left( {com_{n}^{a} ,com_{n + 1}^{a} } \right) = \frac{{\left| {com_{n}^{a} \cap com_{n + 1}^{b} } \right|}}{{\left| {com_{n}^{a} \cup com_{n + 1}^{b} } \right|}}$$

Equation (1) is utilized to ascertain the durability of connections between nodes across communities at various time intervals.

$Community\_Membership$: The ratio of the number of nodes in the interaction between these two communities to the number of nodes in $com_{n}^{a}$ at time n determines the community membership degree of communities $com_{n + 1}^{b}$ at time n + 1 and $com_{n}^{a}$ at time n. This is defined as:

2 $$S\left( {com_{n}^{a} ,com_{n + 1}^{b} } \right) = \frac{{\left| {com_{n}^{a} \cap com_{n + 1}^{b} } \right|}}{{\left| {com_{n}^{a} } \right|}}$$

According to Eq. (2), the extent of community $com_{n + 1}^{b}$ is a member of community $com_{n}^{a} .$ The community membership is used to ascertain the inclusion of one community inside another. More precisely, it may be used to uncover evolutionary occurrences, such as episodes of divergence and fusion. An event is crucial in the understanding of community evolution. The following section offers an elaborate account of evolutionary occurrences.

$Remain$: If the value of community $com_{n + 1}^{b}$ is quite large, a “merge” event takes place, which may be expressed using the following equation:

3 $$rem\left( {com_{n}^{a} ,com_{n + 1}^{b} } \right) = 1\quad if.\exists com_{n + 1}^{b} \in com_{n + 1} ,over\left( {com_{n}^{a} ,com_{n + 1}^{b} } \right) \ge \theta$$

where the term $com_{n + 1}$ refers to a dynamic network at time n + 1.

$Form$: A “form” event occurs if there is very little overlap between community $com_{n}^{a}$ and community $com_{n - 1}^{b}$ at time n-1. This indicates that community $com_{n}^{a}$ at time n is unrelated to any other communities at time n-1. The occurrence may be formalized as

4 $$fo\left( {com_{n - 1}^{b} ,com_{n}^{a} } \right) = 1\quad if.\forall com_{n - 1}^{b} \in com_{n - 1} ,over\left( {com_{n}^{a} ,com_{n - 1}^{b} } \right) < \theta$$

$Disappear:$ When community $com_{n}^{a}$ and $com_{n + 1}^{b}$ have very little community overlap at time n + 1, it means that community $com_{n}^{a}$ at time n is unrelated to other communities at time n + 1. The “Disappear” event that results from this can be depicted using a model.

5 $$disapp\left( {com_{n}^{a} ,com_{n + 1}^{b} } \right) = 1\quad if.\forall com_{n + 1}^{b} \in com_{n + 1} ,over\left( {com_{n}^{a} ,com_{n + 1}^{b} } \right) < \theta$$

$Expand$: If a community at time n, represented by $com_{n}^{a}$, becomes part of another community at time n + 1, represented by $com_{n}^{a} { complement }com_{n + 1}^{b}$, and the number of nodes in $com_{n}^{a}$ is less than the number of nodes in $com_{n + 1}^{b}$, we refer to $com_{n}^{a}$ as “expanding” at time n + 1, as defined by the following formula:

6 $$\exp \left( {com_{n}^{a} ,com_{n + 1}^{b} } \right) = 1\quad iff.\exists com_{n}^{a} ,com_{t} ,1 - \beta \le sh\left( {com_{n + 1}^{b} ,com_{n}^{a} } \right) < 1$$

$Shrink$: A “shrink” event occurs at time n + 1 if the community $com_{n + 1}^{b}$ at time n + 1 is a subset of the community $com_{n}^{a}$ at time n and the number of nodes in $com_{n + 1}^{b}$ is fewer than the number of nodes in $com_{n + 1}^{b}$. This event can be described as follows:

7 $$sh\left( {com_{n}^{a} ,com_{n + 1}^{b} } \right) = 1\quad iff.\exists com_{n}^{a} \in com_{n} ,1 - \beta \le sh\left( {com_{n}^{a} ,com_{n + 1}^{b} } \right) < 1$$

In order to properly identify Expand and Shrink events, the left-hand restrictions for these events with respect to $sh$ are stated as 1-$\beta$, where $\beta \epsilon (\mathrm{0,1}]$. The value of sh has low variability.

$Split$: When $Y=\{{com}_{n+1}^{b},\dots {com}_{n+1}^{b+k}\}$ at time n + 1 has k(< 1) communities, and each community in Y is primarily a part of community ${com}_{n}^{a}$ and the degree of overlap between the union of communities in Y and ${com}_{n}^{a}$ is very high, then ${com}_{n}^{a}$ is said to “split” into distinct communities in the following ways:

8 $$sp\left( {com_{n}^{a} ,Y} \right) = 1\quad iff\left\{ {\begin{array}{*{20}c} {over\left( {com_{n}^{a} ,Y} \right) \ge \gamma } \\ {sp\left( {com_{n + 1}^{i} ,com_{n}^{a} } \right) \ge \gamma \forall com_{n + 1}^{i} \in Y} \\ \end{array} } \right.$$

$Merge$: If there are multiple communities, denoted as $Z = \left\{ {com_{n}^{a} , \ldots com_{n}^{a + k} } \right\}$, at time n, and each community in Z is mostly part of community $com_{n + 1}^{b}$. Additionally, the degree of overlap between the combined set of communities in Z and $com_{n + 1}^{b}$ is significantly high. In such a scenario, an event called “merge” takes place, which can be represented by the following equation:

9 $$sp\left( {com_{n + 1}^{b} ,Y} \right) = 1\quad iff\left\{ {\begin{array}{*{20}c} {over(Z,com_{n + 1}^{b} \ge \beta } \\ {sp(com_{n}^{i} ,com_{n + 1}^{b} \ge \beta \forall com_{n}^{i} \in Z} \\ \end{array} } \right.$$

In Definitions 6–12, the values of θ, γ , and ξ are adjusted via trials to maximize the detection of events.

$Weak\_Shrink$: The occurrence of a minor or quantifiably modest reduction in the number of nodes inside communities is referred to as a weak shrink event. This occurrence coincides with a robust event. Three circumstances can lead to weak shrink events:

- During a Remain event, community $com_{n}^{a}$ transitions to community $com_{n + 1}^{b}$ in the subsequent time period, and the overlapping size between the two communities at different time intervals is less than the size of the communities at time n.
- A Form event occurs when a community at time *n* shrinks in size but does not split, and the event is not noticed even when a new community forms at time n + 1.
- In a Split event, the cardinality of the intersection between community $com_{n}^{a}$ at time *n* and *Y* (which represents the union of communities at time n + 1) is less than the cardinality of $com_{n}^{a}$ at time *n*. The three cases described above can be stated as follows:
  10 $$w_{sh} = \left\{ {\begin{array}{*{20}c} {sh\left( {com_{n}^{a} ,com_{n + 1}^{b} } \right) < 1, \left( {com_{n}^{a} ,com_{n + 1}^{b} } \right) \in rem} \\ {sh\left( {com_{n}^{a} ,com_{n + 1}^{b} } \right) \ge \theta \left\{ {\begin{array}{*{20}c} {\left( {com_{n}^{a} ,com_{n + 1}^{b} } \right) \in fo} \\ {\left( {com_{n}^{a} ,com_{n + 1}^{b} } \right)notequal \in sp} \\ \end{array} } \right.} \\ {sh\left( {com_{n}^{a} ,Y} \right) < 1 \left( {com_{n}^{a} ,X} \right) \in sp} \\ \end{array} } \right.$$

$Weak \_Expand$: A minor proliferation event coexists with major occurrences, suggesting a gradual development in societies. Weak expanding occurrences manifest in the following three situations.

- The number of people in two communities at time n is less than the number of people in the communities at the next timestamp during a Remain event.
- Even if the community at time n expands without a Merge event occurring after a Form event, a new community nonetheless forms at time n + 1.
- At time n + 1, the size of the interaction set between Z and the community ${com}_{n+1}^{b}$ is smaller than the size of ${com}_{n+1}^{b}$ at time n + 1 during a Merge event. The following is a possible representation of the three scenarios given above:
  11 $$w_{exp} = \left\{ {\begin{array}{*{20}c} {sh\left( {com_{n}^{a} ,com_{n + 1}^{b} } \right) < 1, \left( {com_{n}^{a} ,com_{n + 1}^{b} } \right) \in rem} \\ {sh\left( {com_{n}^{a} ,com_{n + 1}^{b} } \right) \ge \theta \left\{ {\begin{array}{*{20}c} {\left( {com_{n}^{a} ,com_{n + 1}^{b} } \right) \in B} \\ {\left( {com_{n}^{a} ,com_{n + 1}^{b} } \right)notequal \in mer} \\ \end{array} } \right.} \\ {sh\left( {Z,com_{n}^{a} } \right) < 1 \left( {Z,com_{n + 1}^{b} } \right) \in mer} \\ \end{array} } \right.$$

$Weak\_Split$: A weak split occurs when a community at time n + 1 is a subset of another community at time n, but the combined communities do not fully reflect the community at time n. This phenomena may be mathematically expressed using the following formula:

12 $$w_{sp} = \left\{ {\begin{array}{*{20}c} {\forall com_{n + 1}^{i} \in Y,sp\left( {com_{n + 1}^{i} ,com_{n}^{a} } \right) \ge \gamma } \\ {over\left( {Y,com_{n}^{a} } \right) < \gamma } \\ \end{array} } \right.$$

$Weak\_Merge$: Weak merge refers to the situation when some communities at time n are part of a single community at time n + 1, but the combined communities do not form the whole community at time n + 1. This phenomena is characterized as follows:

13 $$w_{mer} = \left\{ {\begin{array}{*{20}c} {\forall com_{n}^{i} \in Z,sp\left( {com_{n}^{i} ,com_{n + 1}^{b} } \right)\gamma } \\ {over\left( {com_{n}^{a} ,Z} \right) < \gamma } \\ \end{array} } \right.$$

In Definitions 6–16, the parameter θ is set to a consistent value, as are $\beta$ and $\gamma$. Remark 1: The complexity corresponds to the number of nodes that undergo changes in two communities when events are identified by the framework. The magnitude in the last column denotes the magnitude of the respective communities.

Remark 2: There exists a distinction between the act of dividing and combining events with regards to their temporal sequences. During a splitting event, we evaluate communities at various times in a sequential manner. In contrast, for a merger event, we need to compare communities in a reverse chronological order. Weak events are the result of subtle shifts occurring within societies. Due to the lack of visibility, conventional event-based frameworks are unable to identify these changes. In general, expansive networks undergo gradual evolution via many little alterations, which are hard to identify but yet act as the trigger for significant occurrences. Hence, it may be argued that the detection of subtle occurrences has comparable, if not superior, significance in effectively identifying shifting patterns in dynamic networks. This is crucial in enabling service providers to anticipate future growth of communities. Remark 3: Weak events have a higher frequency relative to strong events, and the presence of several weak events indicates an eventual powerful event.

## Results

The paper conducts a simulation experiment in two steps. Firstly, it verifies the outcomes of tag clustering. Secondly, it evaluates the performance of the proposed novel Community evolution analysis algorithm (NCEA) by comparing it with existing methods such as kg-DFSA^9^ and K-Means Clustering algorithm (KMCA)^10^.

### Simulation setup

With a focus on the MAC sub-layer, the simulation includes several tags and a single reader. It is assumed that there is no noise or interference on the communication path. This assumptions can be focused to validate the theoretical behaviour of the proposed NCEA network under the controlled conditions. When the RFID Network can be deployed in real time, the noise and interference may be introduced and identify quantitatively. When the network topology changed dynamically, the NCEA network identify the weak events based on the persistence and consistency, this is caused by communication noise or interference in RFID system. Additionally, no tags are thought to enter or leave the RFID interrogation zone. We set up a high RFID tag density in this environment, between 100 to 1000 tags. Therefore, during the simulation, the tags were arbitrarily distributed inside the interrogation zone of a single reader.

### Comparative analysis

The metrics used to evaluate performance include accuracy, vulnerability, success rate, delay, and throughputs. The explanation of each measure is provided below.

The accuracy of a network refers to its efficiency in successfully recognizing the number of reader tag communications. This efficiency is measured as a percentage, indicating the accuracy of the network. The accuracy is calculated using Eq. (14),

14 $$Accuracy = 100 \times \frac{vulnerablity}{n}$$

where, $n$ represent the total number of tags.

Vulnerability—During communication, some readers may be unable to access the information contained inside the tags owing to factors such as speed or congestion. The vulnerability of the RFID network is represented by the count referred to as Eq. (15).

15 $$vulnerablity = T_{n} - T_{r}$$

where, $T_{n}$ is total number of tags in network and $T_{r}$ is total number of tags received.

Success rate- The success rate Eq. (16) represents the accurate extraction of tag information by the reader during a certain time period.

16 $$success rate = T_{t}$$

where, $T_{t}$ represents the total count of tags that were read within a certain time interval.

Delay- The entire delay of a packet’s transmission from the source to the destination across a network. The calculation is derived from Eq. (17) below:

17 $$Delay = transmit time - received time$$

Throughput-The calculation is derived from the number of successfully read tags inside the network, as defined by Eq. (18).

18 $$Throughput = \mathop \sum \limits_{x = 1}^{n} read\left( x \right)$$

where x stands for accomplished read tags.

**Fig. 3:**
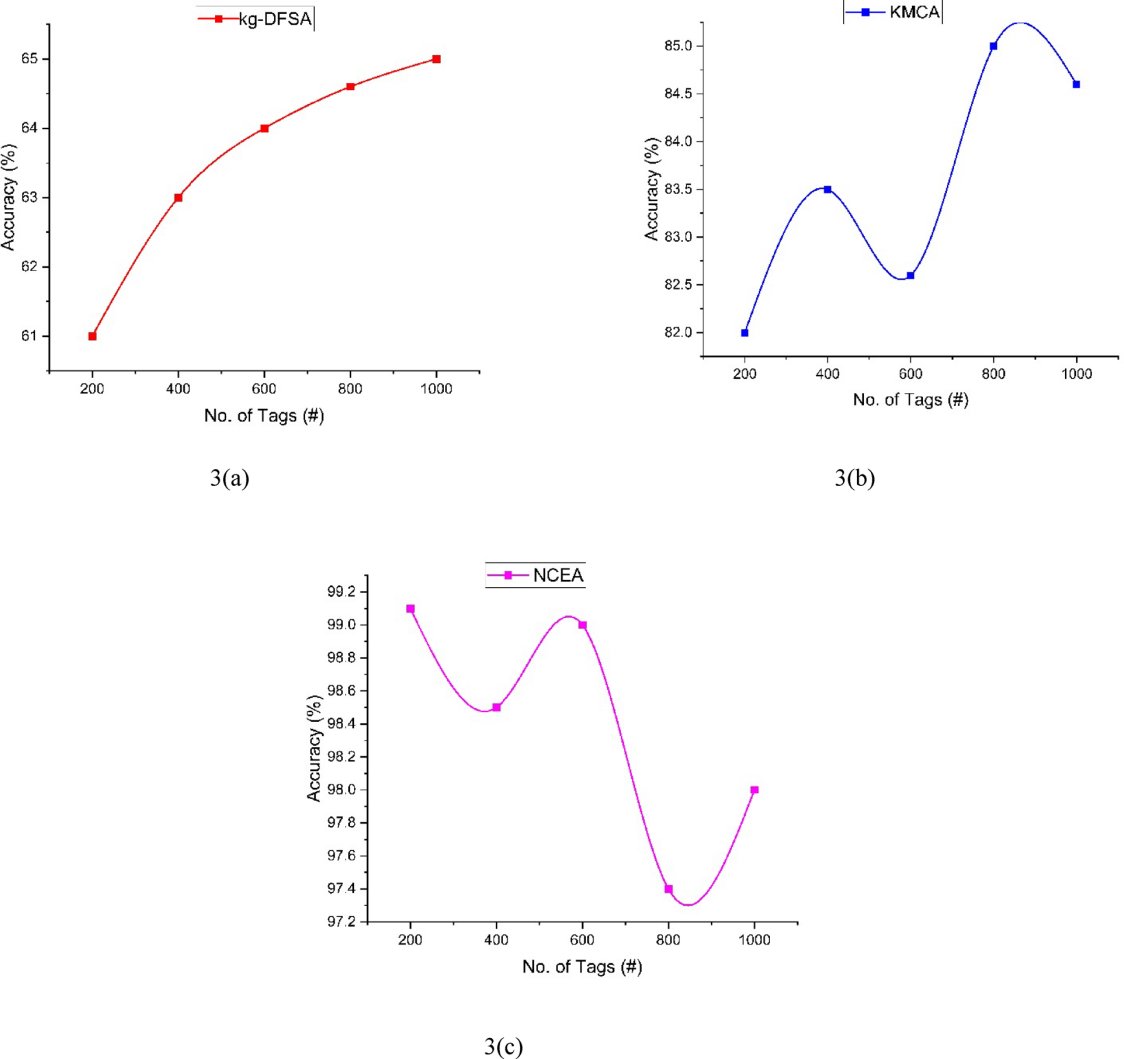
Analysis of Accuracy between kg-DFSA, KMCA, NCEA.

The analysis of accuracy, presented in Table 3 and as well as presented in Fig. 3, provides valuable insights into the performance of three distinct methods: kg-DFSA (3(a)), KMCA(3(b)) and the Proposed NCEA (3(c)). The Accuracy can be compared with three methods kg-DFSA, KMCA, NCEA. The kg-DFSA can achieved 61 to 65% when the tags count is 200 to 1000. KMCA achieved 82% to 85%. When increasing the tags count 400 to 600, the KMCA clustering process slightly decrease the accuracy due to the cluster fluctuations. In particularly, increment the cluster count according to the dense of the tags communication the accuracy increased. NCEA achieved 99.1 to 98%. From the results the NCEA will give better accuracy than the other two methods.

**Table 3:** Comparison of accuracy.

Number of tags	kg-DFSA	KMCA	NCEA
200	61	82	99.1
400	63	83.5	98.5
600	64	82.6	99
800	64.6	85	97.4
1000	65	84.6	98

**Fig. 4:**
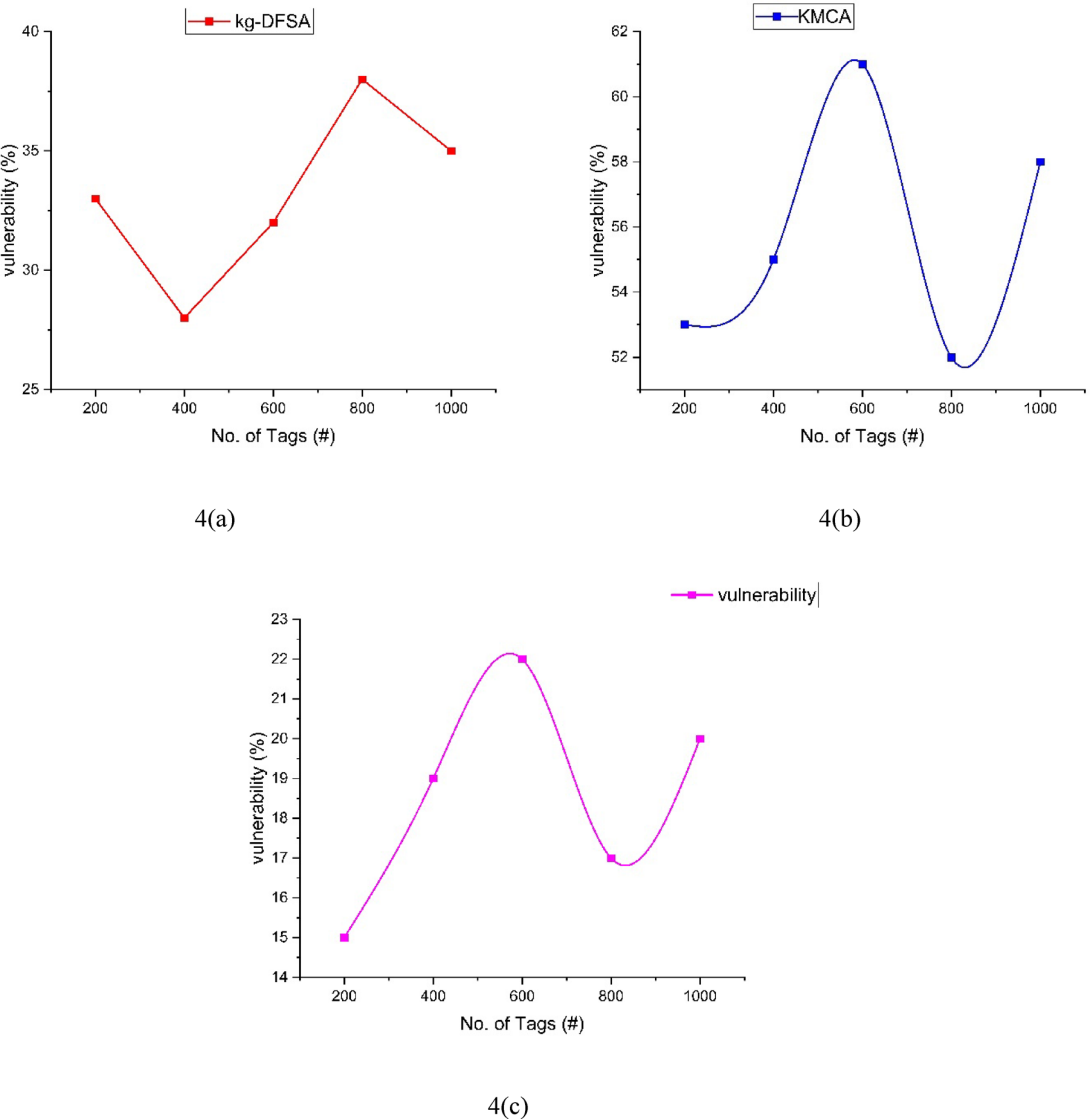
Analysis of vulnerability between kg-DFSA, KMCA, NCEA.

The vulnerability analysis can be illustrated in the Table 4 and Fig. 4. The proposed method NCEA(4(c)) vulnerability can be compared with the other two methods kg-DFSA (4(a)), KMCA (4(b)). From the table, the Proposed NCEA reached the vulnerability at 20%. When the tag count is reached to 1000. KMCA can be achieved 58% vulnerability when the tags count reached to 1000 same way the kg-DFSA can be achieved 35% of vulnerability. Among the three methods kg-DFSA, KMCA and NCEA, the proposed method NCEA have reached only 20%vulnerability. NCEA will perform better than other methods in vulnerability analysis.

**Table 4:** Comparison of vulnerability.

Number of tags	kg-DFSA	KMCA	NCEA
200	33	53	15
400	28	55	19
600	32	61	22
800	38	52	17
1000	35	58	20

**Fig. 5:**
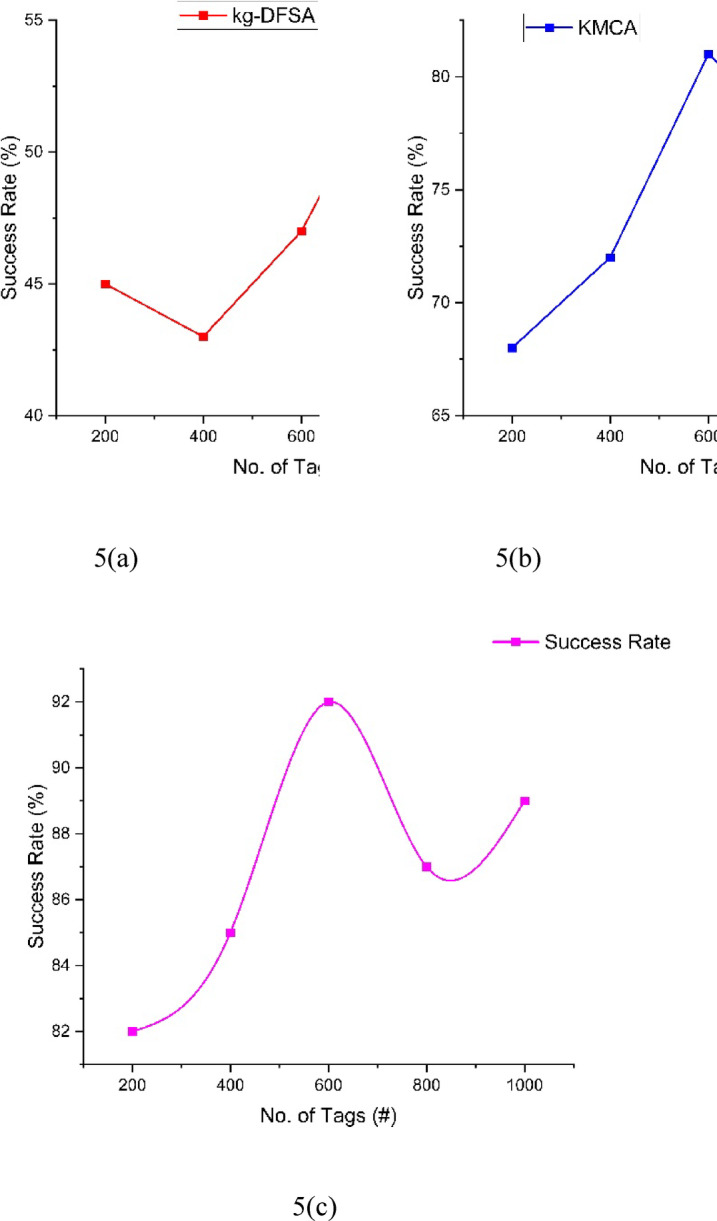
Analysis of Success Rate between kg-DFSA, KMCA, NCEA.

The success rate analysis can be illustrated in Table 5 and Fig. 5. The number of tags can be increased by 200 by every iteration. First iteration 200 tags participated and the success rate for kg-DFSA(5(a)) is 45, KMCA 5(b)) is 68, and the Proposed NCEA (5(c)) is 82. Second iteration, kg-DFSA(5(a)) is 43, KMCA 5(b)) is 72, and the Proposed NCEA (5(c)) is 85, Third iteration, kg-DFSA(5(a)) is 47, KMCA 5(b)) is 81, and the Proposed NCEA (5(c)) is 92, Fourth iteration, kg-DFSA(5(a)) is 54, KMCA 5(b)) is 77, and the Proposed NCEA (5(c)) is 87, Fifth iteration, kg-DFSA(5(a)) is 50, KMCA 5(b)) is 75, and the Proposed NCEA (5(c)) is 89.The proposed NCEA achieved higher success rate among the three methods. Based on the increment of clusters count the success rate may increase.

**Table 5:** Comparison of success rate.

Number of tags	kg-DFSA	KMCA	NCEA
200	45	68	82
400	43	72	85
600	47	81	92
800	54	77	87
1000	50	75	89

**Fig. 6:**
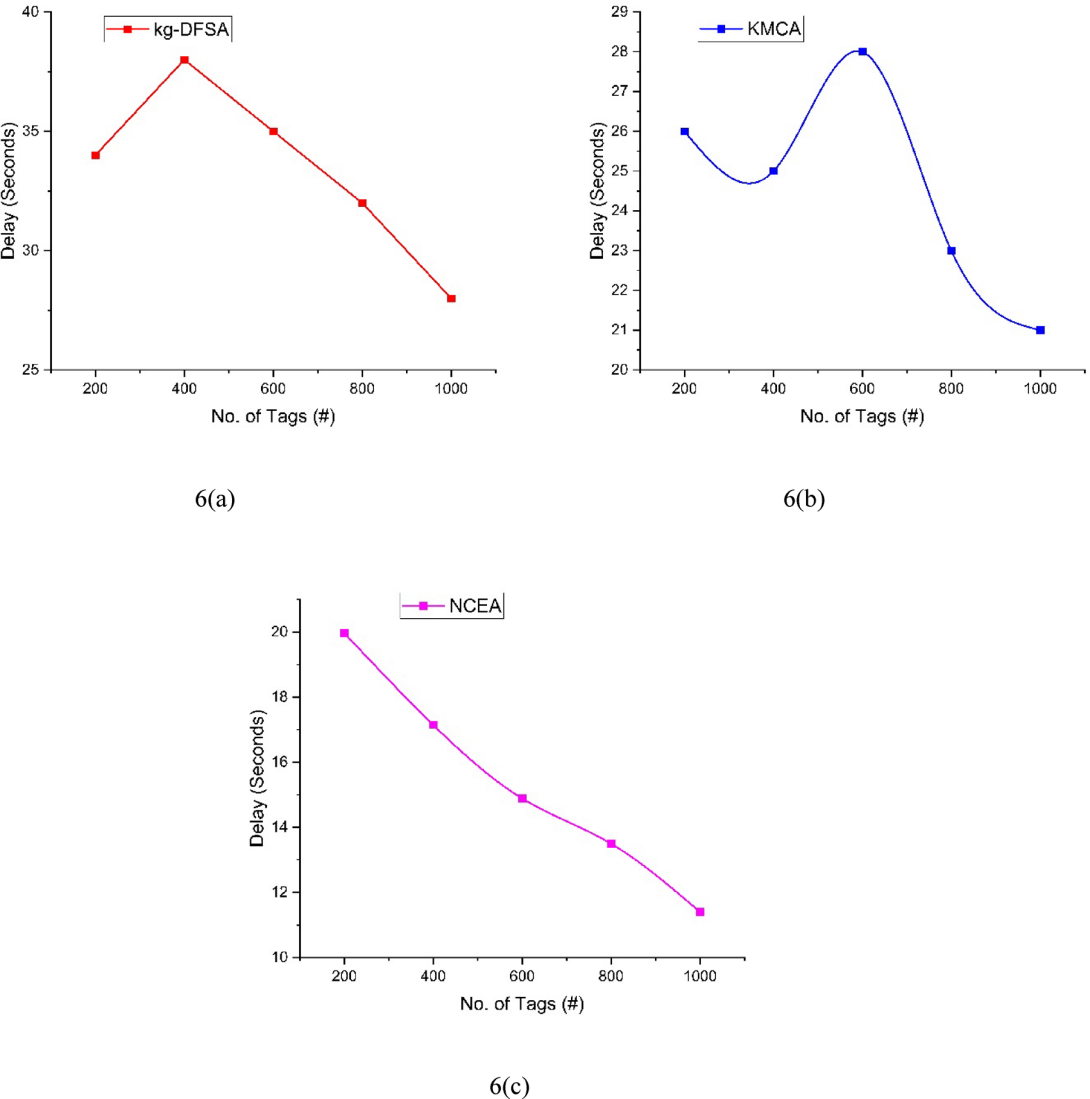
Analysis of Delay between kg-DFSA, KMCA, NCEA.

The analysis of delay can be illustrated in Table 6 and Fig. 6. The number of tags can be increased by 200 by every iteration. First iteration 200 tags participated and the delay for kg-DFSA(6(a)) is 34, KMCA 6(b)) is 26, and the Proposed NCEA (6(c)) is 19.97. Second iteration, kg-DFSA(6(a)) is 38, KMCA 6(b)) is 25, and the Proposed NCEA (6(c)) is 17.15, Third iteration, kg-DFSA(6(a)) is 35, KMCA 6(b)) is 28, and the Proposed NCEA (6(c)) is 14.89, Fourth iteration, kg-DFSA(6(a)) is 32, KMCA 6(b)) is 23, and the Proposed NCEA (6(c)) is 13.5, Fifth iteration, kg-DFSA(6(a)) is 28, KMCA 6(b)) is 21, and the Proposed NCEA (6(c)) is 11.4.The proposed NCEA achieved lower delay among the three methods. Based on the increment of clusters count the delay may decrease.

**Table 6:** Comparison of delay.

Number of tags	kg-DFSA	KMCA	NCEA
200	34	26	19.97
400	38	25	17.15
600	35	28	14.89
800	32	23	13.5
1000	28	21	11.4

**Fig. 7:**
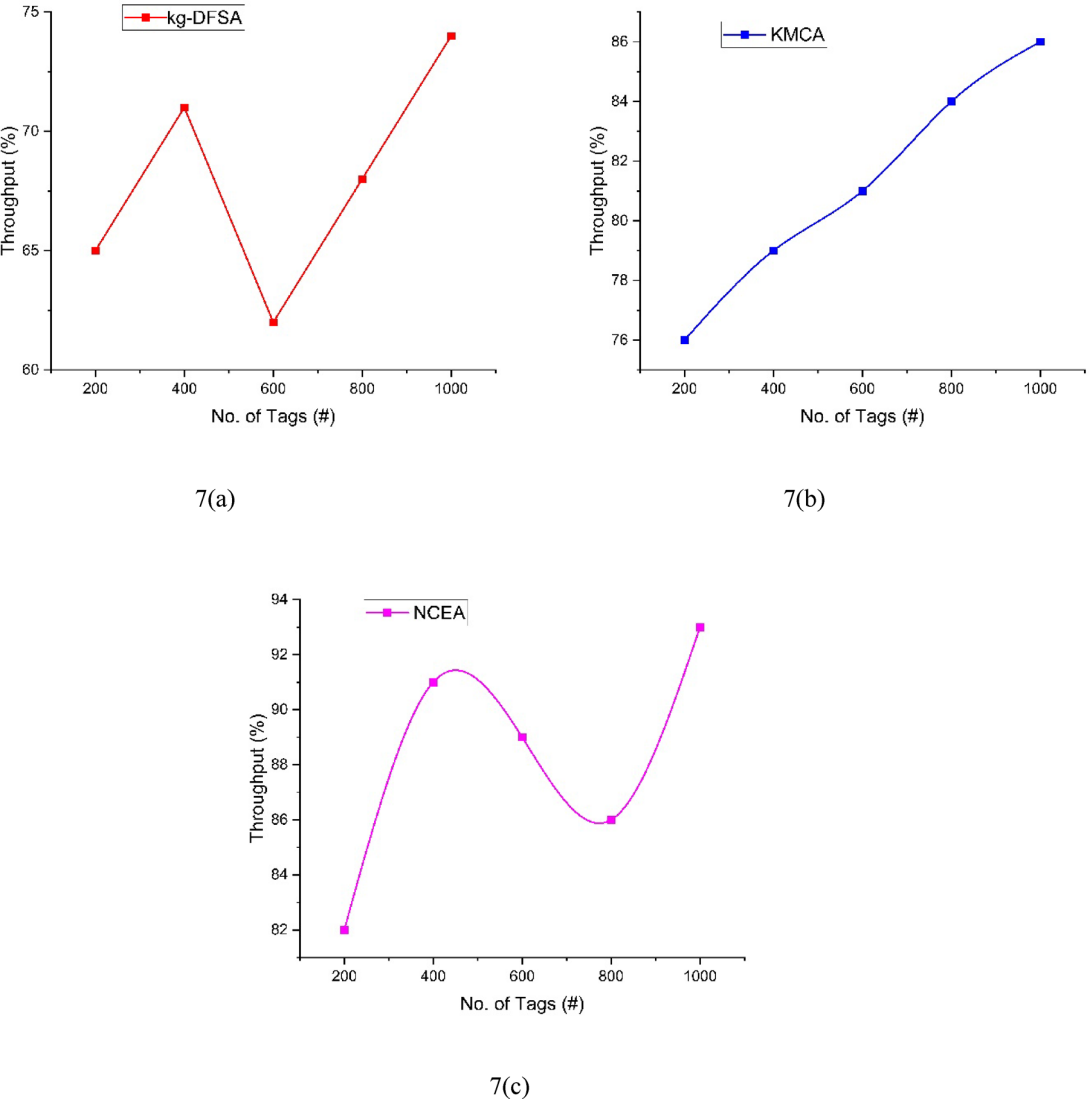
Analysis of Throughput between kg-DFSA, KMCA, NCEA.

The analysis of throughput can be illustrated in Table 7 and Fig. 7. The number of tags can be increased by 200 by every iteration. First iteration 200 tags participated and the delay for kg-DFSA(6(a)) is 65, KMCA 6(b)) is 76, and the Proposed NCEA (6(c)) is 82. Second iteration, kg-DFSA(6(a)) is 71, KMCA 6(b)) is 79, and the Proposed NCEA (6(c)) is 91, Third iteration, kg-DFSA(6(a)) is 62, KMCA 6(b)) is 81, and the Proposed NCEA (6(c)) is 89, Fourth iteration, kg-DFSA(6(a)) is 68, KMCA 6(b)) is 84, and the Proposed NCEA (6(c)) is 86, Fifth iteration, kg-DFSA(6(a)) is 74, KMCA 6(b)) is 86, and the Proposed NCEA (6(c)) is 93.The proposed NCEA achieved higher throughput among the three methods. Based on the increment of clusters count the throughput may increase.

**Table 7:** Comparison of throughput.

Number of tags	kg-DFSA	KMCA	NCEA
200	65	76	82
400	71	79	91
600	62	81	89
800	68	84	86
1000	74	86	93

**Fig. 8:**
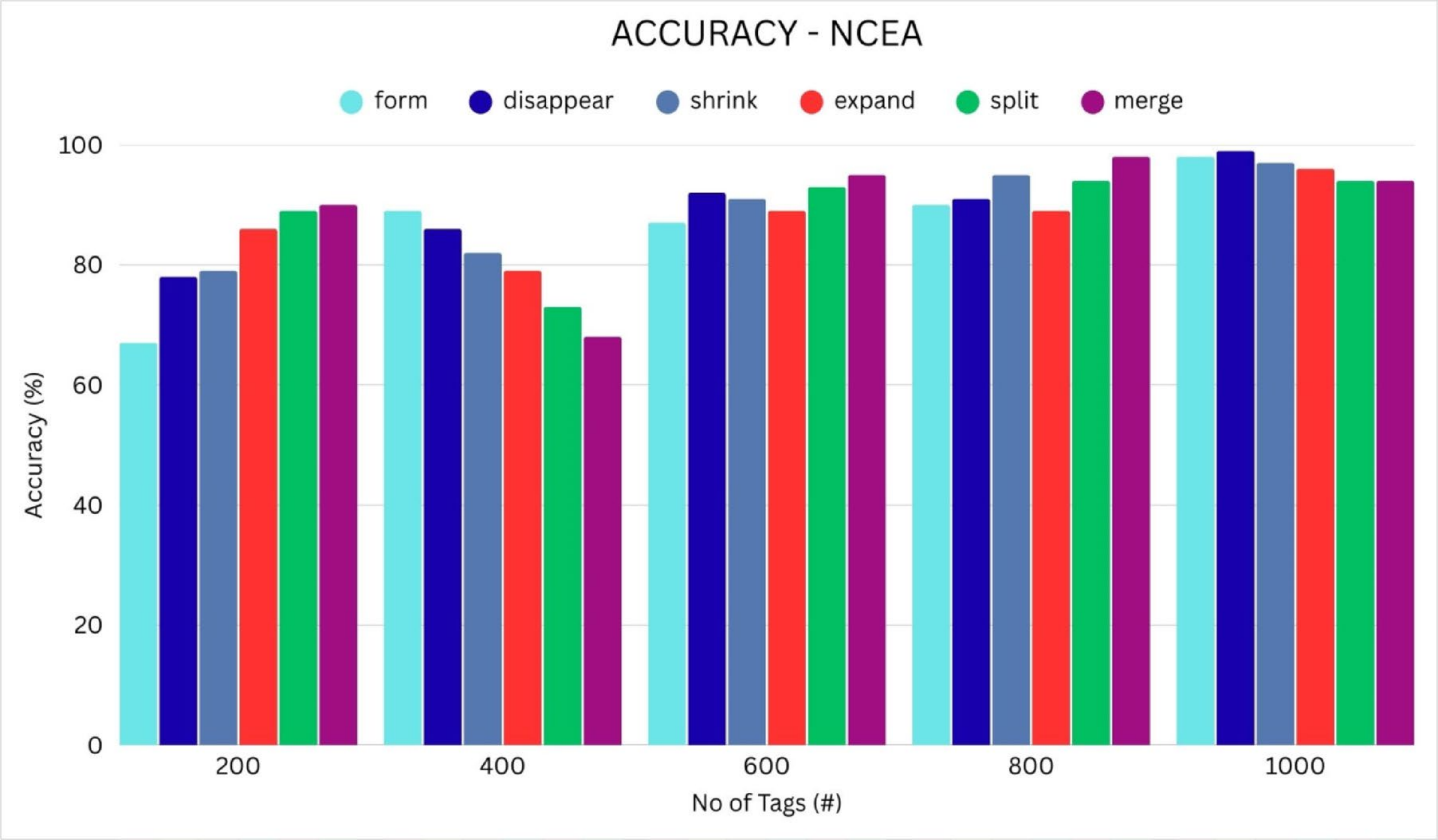
Event detection accuracy.

The analysis of the accuracy of various events is illustrated in Table 8 and Fig. 8. The proposed method demonstrates the accuracy performance of detecting the “disappear” event is 99%, the “form” event accuracy is 98%. “Shrink” event can be achieved 97% and 96% for “expand”. The “split” and “merge” events achieved 94% accuracy.

**Table 8:** Analysis of accuracy for various events.

No of Tags	form	disappear	shrink	expand	split	merge
200	67	78	79	86	89	90
400	89	86	82	79	73	68
600	87	92	91	89	93	95
800	90	91	95	89	94	98
1000	98	99	97	96	94	94

**Fig. 9:**
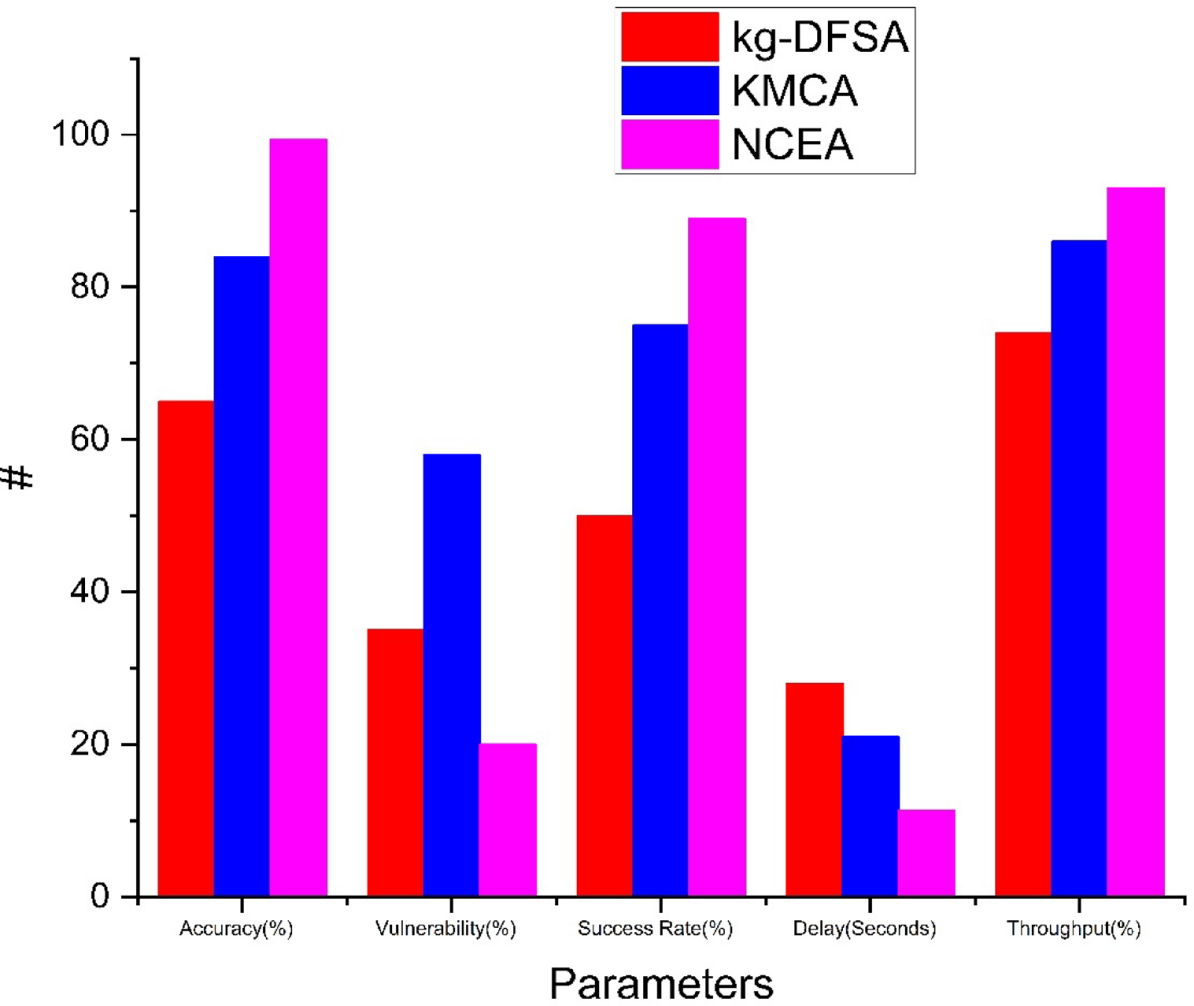
Overall comparative analysis between kg-DFSA, KMCA, NCEA.

Based on the Table 9 the performance of three methods kg-DFSA, KMCA, and NCEA can be illustrated. When the 1000 tags involve in the cluster communication, we can evaluate the performance of the kg-DFSA, KMCA, and NCEA based on some parameters (accuracy, vulnerability, success rate, delay, and throughput.). The accuracy of the proposed NCEA method achieved 99.4% high reliability kg-DFSA 65% and KMCA 84%. The vulnerability of the network is NCEA: 20, kg-DFSA: 35 and KMCA: 58. Success rate achieved by NCEA: 89, kg-DFSA:50 and KMCA:75. Delay of the NCEA method is 11.4, kg-DFSA is 28 and KMCA is 21. The high throughput can be achieved by NCEA:93%, other kg-DFSA: 74, KMCA:86. Figure 9 represents the overall comparative analysis of kg-DFSA, KMCA, NCEA.. Among all the three methods, the proposed NCEA performed well compare than the other methods.

**Table 9:** Overall comparative analysis.

Parameters	kg-DFSA	KMCA	NCEA
Accuracy (%)	65	84	98
Vulnerability (%)	35	58	20
Success rate (%)	50	75	89
Delay (sec)	28	21	11.4
Throughput (%)	74	86	93

## Conclusion

This research can be supported to construct a road map with RFID support and without interference of satellite. Based on the outcome of this research, the proposed method Novel Community Evolution Analysis performs well for cluster head selection based on the energy and neighborhood metrics of the participated nodes in the cluster network. This research proves, the proposed method performed well than kg-DFSA, KMCA in terms of different parameters such as Accuracy, delay, vulnerability, success rate and throughput. The continuity of dynamic network in RFID clustered networks is assessed using two metrics: network membership degree and network overlapping degree. The research results have shown the efficiency of NCEA method for calculating the network overlapping degree and network membership degree. When enlarge the network size and increase the nodes count in RFID network the NCEA proves the efficiency by the results. Furthermore, the experimental results have also shown the efficiency of the NCEA framework in recognizing both robust and weak occurrences. It has been noticed that the proposed NCEA method demonstrates an accuracy of 98%, a vulnerability rate of 20%, a success rate of 89%, a latency of 11.4 s, and a throughput of 93%. These results highlight the effectiveness of our method in enhancing the energy efficiency and overall performance of large-scale RFID networks. In future research, the large-scale RFID network will support to adopt the IoT devices and VANET. The simulation results were made to assume a noise free network environments. The NCEA network is inherently robust the real time disturbances. Particularly, weak event identification method filters the network topology changes frequently, which is caused by communication noise or interference in RFID systems.The extended version of this research, the experimental results evaluation analysis can be incorporate explicitly noise and interference models to further assess the real time RFID network.

## Data Availability

The simulations produced the data used in the present study, and all simulation datasets and scripts used to produce them can be obtained from the corresponding author upon request, provided a reasonable request is made.
